# Optimal sampling of spatial patterns improves deep learning-based early warning signals of critical transitions

**DOI:** 10.1098/rsos.231767

**Published:** 2024-06-05

**Authors:** Smita Deb, Ekansh Mahendru, Paras Goyal, Vishwesha Guttal, Partha Sharathi Dutta, Narayanan C. Krishnan

**Affiliations:** ^1^ Department of Mathematics, Indian Institute of Technology Ropar, Rupnagar, Punjab 140001, India; ^2^ Department of Computer Science, Indian Institute of Technology Ropar, Rupnagar, Punjab 140001, India; ^3^ Centre for Ecological Sciences, Indian Institute of Science Campus, Bengaluru, Karnataka 560012, India; ^4^ Department of Data Science, Indian Institute of Technology Palakkad, Palakkad, Kerala 678623, India

**Keywords:** spatial patterns, catastrophic and non-catastrophic transitions, tipping points, deep learning, early warning indicators, classification

## Abstract

Complex spatio-temporal systems like lakes, forests and climate systems exhibit alternative stable states. In such systems, as the threshold value of the driver is crossed, the system may experience a sudden (discontinuous) transition or smooth (continuous) transition to an undesired steady state. Theories predict that changes in the structure of the underlying spatial patterns precede such transitions. While there has been a large body of research on identifying early warning signals of critical transitions, the problem of forecasting the type of transitions (sudden versus smooth) remains an open challenge. We address this gap by developing an advanced machine learning (ML) toolkit that serves as an early warning indicator of spatio-temporal critical transitions, Spatial Early Warning Signal Network (S-EWSNet). ML models typically resemble a black box and do not allow envisioning what the model learns in discerning the labels. Here, instead of naively relying upon the deep learning model, we let the deep neural network learn the latent features characteristic of transitions via an optimal sampling strategy (OSS) of spatial patterns. The S-EWSNet is trained on data from a stochastic cellular automata model deploying the OSS, providing an early warning indicator of transitions while detecting its type in simulated and empirical samples.

## 1. Introduction

Bifurcation-induced transitions are observed in various complex systems under adverse conditions such as in human societies [1], disease in organisms [2–4], palaeoclimatic systems [5,6], lake and forest habitats [7,8] and other bio-ecological systems [9,10]. Studies also provide evidence of the association of spatial patterns with an approaching bifurcation [11,12]. At the bifurcation point, the system undergoes either a sudden (catastrophic) shift to an alternative stable state, also known as a critical transition, or the system can experience a smooth (non-catastrophic) change in the state variable depending on the kind of bifurcation. While the former is associated with a first-order or discontinuous transition via a saddle-node/fold bifurcation aka critical transition in the nonlinear dynamics/ecological community, the latter occurs whenever the system undergoes a second-order or continuous transition via other forms of bifurcations (e.g. transcritical, pitchfork and Hopf bifurcations) [13]. Structural changes are envisioned in the system dynamics with variation in the control parameter as the bifurcation point is crossed. While both the catastrophic and the non-catastrophic transitions incur loss and need to be prevented, sudden shifts to undesirable states demand predictions and focused mitigation. Owing to such concerns, classifying the type of transition before the transitions occur is of significant importance across interdisciplinary fields [14–16].

Investigation of spatial statistical properties such as spatial autocorrelation function, spatial variance and other higher order moments of the state variables across space to forewarn transitions have been shown effective in both theoretical and empirical studies [11,17–22]. These indicators arise from the phenomenon of critical slowing down, where a local perturbation takes longer to recover when the system is in the vicinity of a critical point. As a consequence, nearby cells become increasingly similar to one other, thus leading to increased spatial autocorrelations. Furthermore, owing to slowed recovery, the system also exhibits stronger fluctuations, leading to increases in quantities such as spatial variance and higher moments. As the system approaches the tipping point, it takes longer to recover to the equilibrium point upon perturbation. This phenomenon is termed critical slowing down (CSD). Although this is a critical feature before transition, this phenomenon arises in both sudden and smooth transitions [23], on account of which the CSD-based generic early warning signals (EWS) fail to classify the type of a transition. Furthermore, stochasticity can interfere with the strength of the critical slowing down signals [24,25]. Therefore, there is a need to improve our existing toolboxes of EWS that can overcome these limitations.

With the development of modern tools for improved detection of transitions being obligatory, machine learning (ML) methods are the front-runners as they tackle complex data in an automated course of action. Furthermore, ML models are known to capture latent features and hidden patterns from raw data and have widespread utility in tasks involving the classification and regression of high-dimensional datasets. However, thus far, there has been a limited application of ML methods in the context of EWS [14,15]. These recent studies have developed deep learning-based models to forecast critical transitions from pre-transition time series data. Of note, these studies take a step ahead in discerning types of bifurcation in time series generated from mathematical models and empirical data. However, questions such as whether we can classify transitions from sequences of spatio-temporal patterns [26] and whether we can predict the distance from the tipping [27] are only recently addressed with room for further ameliorations.

Here, we aim to develop an ML-based EWS of transitions in spatial systems from spatio-temporal patterns, particularly overcoming the issues when sampling from raw data only without any preprocessing by implementing a novel optimal sampling strategy (OSS). Here, we propose a spatial EWS network (S-EWSNet) composed of time-distributed convolutional neural network (CNN) and long- and short-term memory (LSTM) sub-modules. While it is apparently unclear whether the ML model learns from densities or other system-specific patterns, it is important to ensure this is not the case. With evidence that density gradients across labels (transition classes) are an intruding feature preventing the model from capturing the essential features but a change in density, we implement a novel optimal sampling strategy, namely OSS. This sampling technique for input sequences shall ensure the generality of the S-EWSNet while compelling the model to learn the characteristic features delineating the respective transition types. Furthermore, to account for the robustness of S-EWSNet, we consider simulated data from a different origin than training data, alongside testing on a few available empirical data. With minor data preprocessing and no presumptions on the underlying system, S-EWSNet is an application of ML with the ability to classify diverse bifurcation-induced transitions in real data across cross-disciplinary fields.

## 2. Models and methods

### 2.1. Spatially explicit models and data generation

Below, we discuss the details of two spatial models exhibiting sudden and smooth transitions. One of the spatial models is deployed for training (hereafter termed the ‘training model’), and the other one is used for testing (termed the ‘testing model’) the ML model. Data corresponding to two labels: ‘sudden transition’ and ‘smooth transition’, are generated from each spatial model.

**Figure 1.**
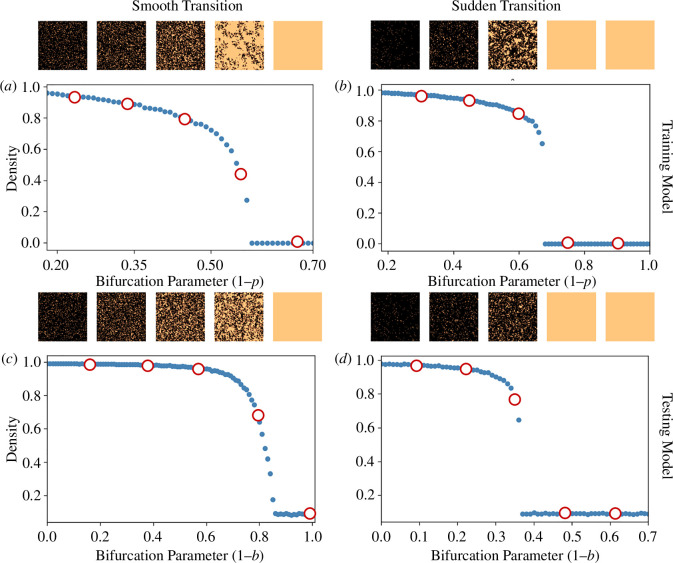
Catastrophic (sudden) and non-catastrophic (smooth) transitions in the training and testing models: Spatial mean density plotted against gradual change in the bifurcation parameter, exhibiting (*a*) smooth (*q* = 0.7) and (*b*) sudden transitions (*q* = 0.8) used for training, and (*c*) smooth (*f* = 0.8) and (*d*) sudden (*f* = 0.1 ) transitions used for testing S-EWSNet. Spatial patterns are plotted above respective transition scenarios. Red circles indicate the locations of spatial patterns along gradients of the bifurcation parameter from left to right. Changes in spatial patterns are observed along the transition/bifurcation gradient.

The dataset for *training* the deep learning model is generated using a cellular automata model [17,22] exhibiting smooth (figure 1*a*) and sudden (figure 1*b*) transitions depending on the strength of positive feedback q with variation in driver parameter p . The dynamics of the training model can be well explained by the below set of birth–death processes,


(2.1) $$\begin{array}{rl} & 01\to 11=10\to 11=p,\\ & 01\to 00=10\to 00=1-p,\\ & 11\to 01=11\to 10=(1-p)(1-q),\\ & 110\to 111=011\to 111=q, \end{array}$$

where 0 and 1 are the possible entries in a cell and imply an unoccupied and an occupied cell, depending on the probabilistic update rule for states of the system. Entries in each randomly chosen grid cell are updated with a probability p of local establishment and considering the influence of local positive feedback using a parameter q . The parameter q enhances the probability of birth and death depending on whether the neighbouring cells are occupied or unoccupied, respectively, and the change in q value consequently determines whether the system undergoes a sudden ( q≥0.85 ) or a smooth transition ( q≤0.8 ). We consider a two-dimensional lattice of N×N grid for simulation. Further details of the cellular automata model are presented in electronic supplementary material, appendix, S1 and the algorithm used for updating cells in the two-dimensional lattice is summarized in electronic supplementary material, appendix, algorithm A1 [22].

For *testing* the generalizability of the deep learning model, we consider the local facilitation model [9]. Data from this testing model are generated from stochastic simulations the same manner as the training model using algorithm A1 (see electronic supplementary material, appendix) and exhibit both types of transition (see figure 1*c*,*d*) for different local facilitation rate ( f ). The above model is representative of an arid ecosystem and contains three possible states (occupied, degraded and unoccupied). For our purpose, we map occupied cells as 1 and both degraded and unoccupied as 0 . The transition probability from one state to the other for this model is as follows:


(2.2) $$\begin{array}{l} w_{\left[0,+\right]}=\delta \rho_{+}+(1-\delta {)}_{q+|0}(b-c\rho_{+}),\\ w_{\left[-,0\right]}=r+fq_{+|-},\\ w_{\left[+,0\right]}=m,\\ w_{\left[0,-\right]}=d, \end{array}$$

where $w_{\left[0,+\right]}$ denotes the probability of colonizing an unoccupied cell, $w_{\left[-,0\right]}$ is the probability of regeneration of a degraded cell, $w_{\left[+,0\right]}$ represents the probability of mortality of an occupied cell and $w_{\left[0,-\right]}$ is the probability of degradation of an unoccupied cell. δ is the fraction of globally dispersed seeds, $\rho_{+}$ is the abundance of vegetated state and $q_{j|i}$ is the probability of finding the neighbour of a cell in a j th state ( j∈{0,+,−} ). b is the intensity of environmental deterioration, c is the net effect of intrinsic seed production and competition owing to global density, r is the probability of regeneration of a cell lacking any neighbouring occupied cell, f is the facilitation rate, m is the mortality probability of an occupied cell, and d denotes the degradation probability of an unoccupied cell. For spatial simulations of both the models, we consider isotropic lattice, that is, no particular direction is followed to cover all the N×N grids of the lattice. Ground truth labels (or the type of transition) are assigned to the data for the considered parameter regimes by referring to previous literature [9,22].

For the data generation, corresponding to the third label ‘no transition’ from both the training and testing models, we sample the sequences of data at different fixed values of the bifurcation parameter. However, the average density is kept similar to the data corresponding to the other two labels to persuade S-EWSNet to learn more complex features, ignoring the range of densities as an attribute to distinguish the transitions. Spatial bootstrapping, which samples data with replacement randomly so that any correlations in time or space are removed can also be used for generating samples corresponding to the no transition label from raw snapshots. We use an equivalent number of samples corresponding to each label for training the deep learning model.

### 2.2. Data preprocessing to ensure generality

**Figure 2.**
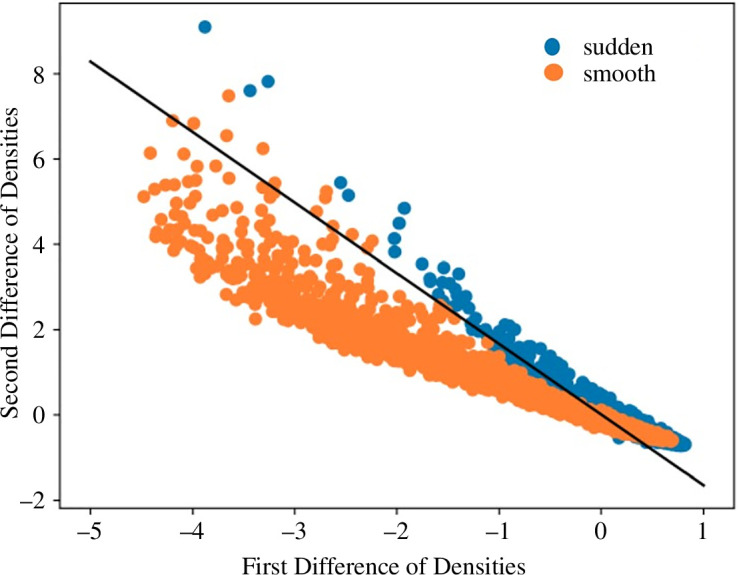
Separation of the transition class labels using first and second differences in densities: sudden (catastrophic) and smooth (non-catastrophic) transition classes are linearly separable using first and second differences in densities of sequences of snapshots. Snapshots are sampled uniformly in time or linearly across control parameters, adding to easier identification and lack of generalizability.

Conducting preliminary experiments shows that the classes of sudden and smooth transitions are mostly distinguishable just by using the first and second differences in the densities of the snapshots to the point that they are linearly separable (see figure 2). This is owing to the fact that the snapshots are sampled uniformly in time, and sudden shifts where the changes are discontinuous would have larger first and second derivatives near the tipping point. Using these features for early predictions assumes that the driver/bifurcation parameter changes linearly with time, which may not be the case in the real world. The abrupt change of states in the case of sudden transitions also causes the sequences to contain mostly the initial stable state if sampled for a linear increase in the control parameter. This creates another challenge since the stable states far away from the tipping contain less information about the upcoming transition. Such sequences are almost indistinguishable between the classes. The former issue will cause an ML model to learn system-specific feature values, while the latter will lead it to underperform. These issues need to be remedied to ensure that features related to the bifurcation types are captured by the model. Methods like detrending the data or obtaining residuals after smoothing using various smoothing filters may be useful methods for preprocessing data from continuous partial differential equations, but are likely to be associated with manual interventions and preprocessing errors analogous to the temporal counterpart. However, if the underlying data are discrete (binary as in our case), it does not work. This demands the need for a sampling strategy that shall overcome all the aforementioned issues while being computationally efficient. Furthermore, the ML model trained over an optimally sampled training set can be applied to sequences from different provenances.

### 2.3. Optimal sampling strategy

**Figure 3.**
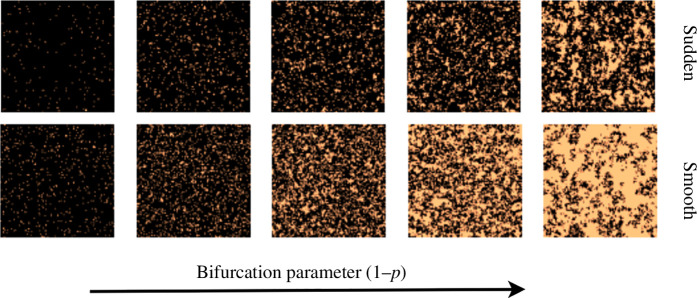
Example sequence of patterns sampled using OSS: sequence of pre-transition patterns obtained along an increase in bifurcation parameter for sudden (upper panel) and smooth (lower panel) transitions in the training model.


**Figure 4.**
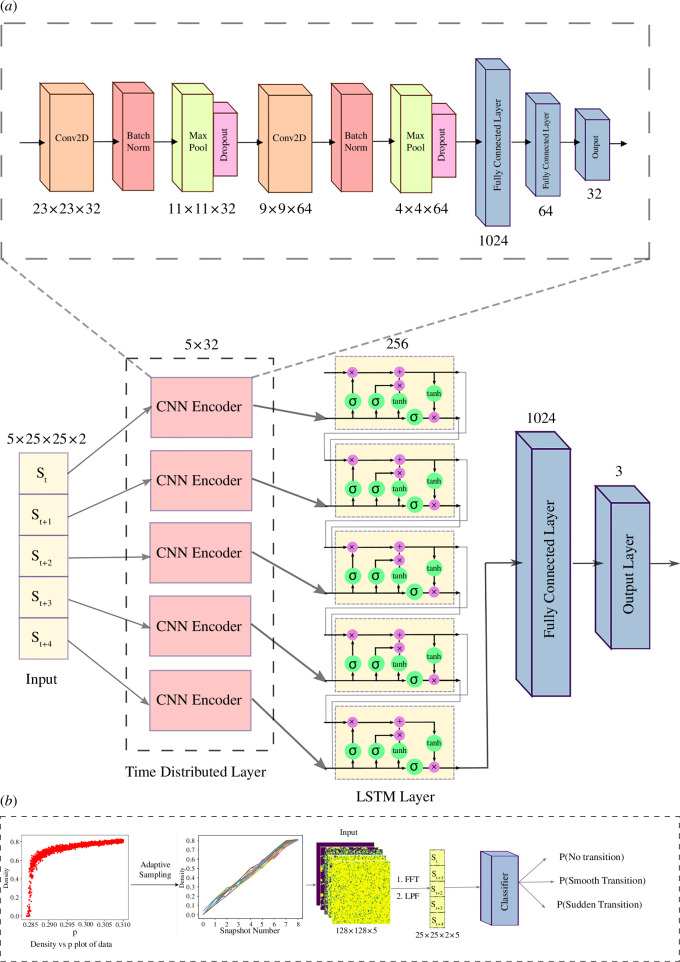
S-EWSNet architecture and overall model workflow: schematic diagram for S-EWSNet composed of CNN and LSTM layers and a final output layer to classify a transition type from input sequences of pre-processed data (*a*). The flowchart shows all the steps for the classification of transitions by S-EWSNet. Sequences of snapshots are sampled corresponding to the three labels (no transition, smooth transition and sudden transition) and are then adaptively resampled using OSS and passed through convolution blocks and LSTM layers. The weights from the trained model, S-EWSNet, now return the prediction probability corresponding to classified labels for any test instance (*b*).

Both the issues, sampling data far away from a tipping point and linear separability of transition types from trends in first- and second-order differences, mentioned in the previous section, are solved by adopting our optimal sampling technique, termed OSS. With this technique, the sampling must be such that it prevents any information about the control parameter from leaking into the input sequences, which the ML model would otherwise use to distinguish between the classes. Here, we address the above issues by sampling snapshots on the densities rather than the control parameter. The generated snapshots (images) are sorted into buckets based on the density of the snapshot. The input sequences are generated by sampling one snapshot from each bucket. Now the sequence has no information about the control parameter apart from how the spatial patterns evolved overdensities (see figure 3 and the flowchart in figure 4). However, following this OSS, one must generate the sequences before adding them to the bin. We ensure sufficient snapshots in each bin by using differential sampling on the control parameter where a finer resolution is chosen near the tipping point than further away from it. Thus, the OSS automatically prevents the model from learning from differences in densities across class labels, as sequences of snapshots corresponding to each class label now have similar differences in densities. This also addresses the second issue mentioned in the above section, allowing snapshots closer to the tipping to be included in the input sequence. This is essential as changes in the system undergoing a transition are more prominent closer to the tipping, particularly for systems undergoing sudden or critical transitions. The algorithm for sampling input sequences is as follows:

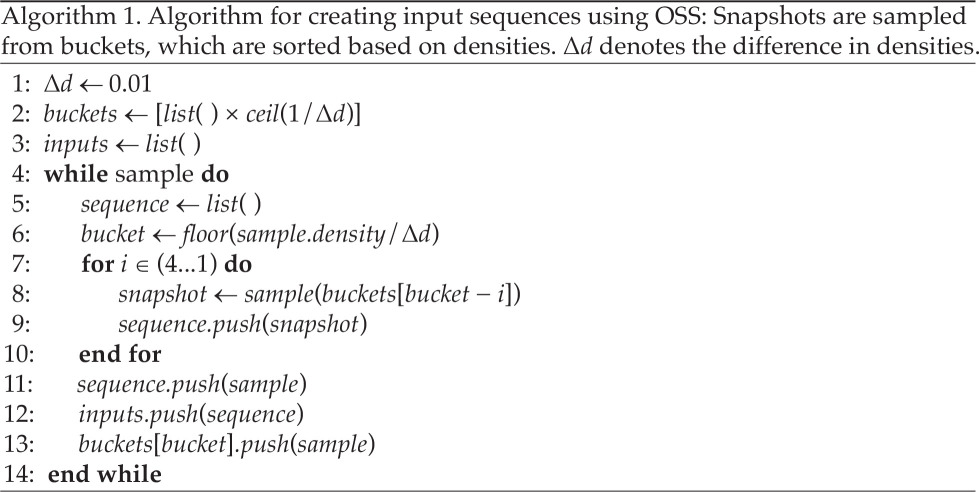


As evident from our analysis in the previous section, raw spatial snapshots considered for training the ML model will overfit and can hamper the generalizability of the model. In addition to the OSS (see algorithm 1) to tackle this issue, we add another check by sampling the lower frequency from the Fourier transform of the raw snapshots. While it is beyond the scope to apprehend the decision of the ML-based early warning indicator, this additional step of employing the Fourier transform is intended to enable the ML model to learn only from the necessary information and bypass density-related features. Carpenter and Brock [28] and Kefi *et al*. [11] have previously confirmed the efficiency of discrete Fourier transform in deriving indicators showing trends before bifurcation-induced transitions. A peak at the lower frequencies is seen in the case of these bifurcations. Thereby, we clip the Fourier transform of each snapshot in the input sequence from a size of 128×128 to 25×25 around the lower frequency components to obtain the centre peak as the input to the model. The intuition behind this step is to remove the system-specific high-frequency data that might mislead the ML model while learning attributes specific to the model and not bifurcations.

### 2.4. Deep learning model: S-EWSNet

S-EWSNet in figure 4 is built on a CNN-LSTM) architecture. A CNN [29] encoder is time-distributed, consisting of stacked convolutional blocks, each composed of convolutional, batch normalization and rectified linear unit (ReLU) activation layers. The resultant operation after one stacked convolutional block appears as follows:


Z=ReLU(BN(W∗X)),

where ∗ is the convolutional operator, *BN* denotes batch normalization and ReLU is the rectified linear unit activation function. To make S-EWSNet invariant to the input dimension, we apply a max-pooling operation, and this is followed by a dropout layer, an additional check to avoid overfitting. The final output from the CNN is obtained after passing through fully connected layers, which allow learning combinations of all features from the previous layer followed by a dense layer. The output from the CNN is then fed into a LSTM [30] layer, which then feeds into the output dense layer. This is followed by a softmax activation to return the output as a classification label. LSTM is a recurrent neural network found effective in learning trends over long input data sequences. The LSTM is composed of the forget gate ( $f_{t}$ ), parametrized by $w_{f}$ , which decides the information to be deleted from the cell state ( $C_{t}$ ) on obtaining the input ( $X_{t}$ ) at time t and can be defined as follows:


$${\text{f}}_{t}=\sigma (<{\text{w}}_{f},[{\text{H}}_{t-1},{\text{X}}_{t}]>),$$

where σ is the Sigmoid activation function and <.> is the dot product operation. The input gate ( $i_{t}$ ), parametrized by ( $w_{i}$ ), determines the information that should be added to the cell state $C_{t}$ and is defined as


$${\text{i}}_{t}=\sigma (<{\text{w}}_{i},[{\text{H}}_{t-1},{\text{X}}_{t}]>).$$

The cell state $C_{t}$ is obtained by using both $f_{t}$ and $i_{t}$ in the following manner:


$$C_{t}=\tanh\left(<w_{c},[H_{t-1},X_{t}]>\right),$$

(2.3) $$C_{t}=f_{t}⊙C_{t-1}+i_{t}⊙{\tilde{C}}_{t},$$

where $w_{c}$ parametrizes the intermediate cell state and ⊙ is the Hadamard product. The hidden state $H_{t}$ and output gate $o_{t}$ (parametrized by $w_{o}$ ) of the LSTM are defined as


(2.4) $$o_{t}=\sigma \left(<w_{o},[H_{t-1},X_{t}]>\right)and$$

(2.5) $$H_{t}=o_{t}⊙\tanh(C_{t}).$$

S-EWSNet comprises stacked convolutional blocks feeding into an LSTM block with 256 hidden units (see figure 4 for more details).

As the input is made up of complex numbers from the Fourier transform, there are two channels for each pixel in the spatial data: namely, the magnitude and the angle of the complex number. A time-distributed CNN allows us to pre-process the input sequence before actually passing it to the LSTM cells. LSTM cells cannot directly process the input matrix. For processing the matrix, we use convolutional layers, and a network of these layers is applied to each matrix in the input sequence. Next, the intermediate results are fed to the LSTM layers, followed by dense layers that finally return the classified labels corresponding to the input matrices pertaining to sequences of snapshots. Using the time-distributed layer, each input matrix gets processed independently, and the data are finally processed as a sequence from the LSTM and dense layers. The input to S-EWSNet is the sequence of clipped Fourier transform data generated from a sequence of pre-transition spatio-temporal data sampled uniformly on the average density of the snapshots from the training model using the above-mentioned algorithms. In the end, the model outputs labels corresponding to the ‘sudden transition’, ‘smooth transition’ and ‘no transition’ classes on passing sequences of snapshots.

## 3. Results

### 3.1. Detecting transitions using S-EWSNet

**Figure 5.**
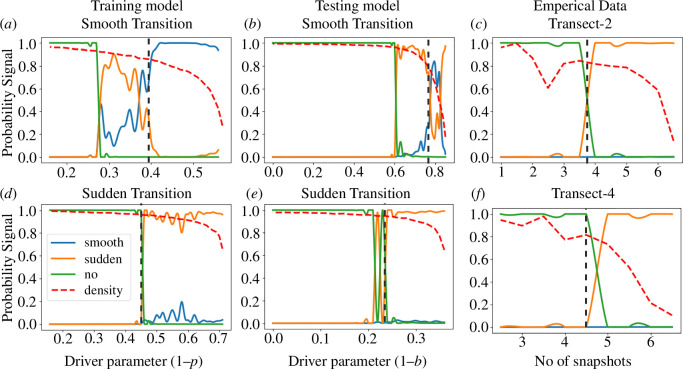
Trends in S-EWSNet indicator on test instances: Probability signals as obtained from S-EWSNet for simulated data corresponding to both transition (sudden and smooth) labels from the (*a*) and (*d*) training model (test instances obtained from equation (2.1) but are different to that of training samples), (*b*) and (*e*) testing model equation (2.2), and (*c*) and (*f*) empirical samples from Savanna ecosystem. In all the instances, we obtain the prediction probability for the type of transition or no transition. We observe that the dominant probability signal is obtained for the correct transition type. The red dashed curve represents the mean density of the snapshots along the bifurcation gradient. The vertical grey dashed line in each sub-figure marks the region after which the correct signal is dominant throughout. Clearly, in all the instances, the dominant probability is obtained for the respective transition type.

Guided by the efficiency of deep neural networks in a wide range of complex classification tasks, we investigate the results obtained after training S-EWSNet (CNN-LSTM deep neural network) on data generated using OSS. The deep neural network S-EWSNet was trained using the clipped Fourier transforms of sequences of snapshots sampled from the training model, equation (2.1). The sampling method ensures that in each iteration, a new sequence of snapshots is generated, allowing us to validate the model robustness on data generated from the training model. We test on instances, each being a sequence of length five starting from the highest density, that is, high p value ( p=0.8 ). We run the model on this sequence and then delete the first snapshot and add another snapshot of lower density to the end of the sequence. We keep on repeating this process till the tipping point is reached (the mean vegetation cover drops to 0 for the first time; this sample is excluded from the input sequence). The confidence values from S-EWSNet work as an EWS of critical transitions, showing an increasing trend in the prediction probability prior to the transition. The model also discerns such transition from smooth transition or no transition. While testing, the ML model exhibits good results when a minimum of five snapshots are available. The S-EWSNet can classify the type of transition well before the actual point of transition is reached, as observed in figure 5.

We further test S-EWSNet on data generated from the testing model, equation (2.2). The S-EWSNet showed a positive correlation with the expected results. Figure 5*c*,*d* highlights the model performance for both the transition labels. For the initial part of the pre-transition data, the S-EWSNet confuses between the transition types as evident from the fluctuations in the prediction probabilities corresponding to the different labels/transition types. However, approaching closer to the bifurcation point, the predictability of S-EWSNet improves, and it signals the type of transition with higher prediction probability. In this model, densities are distributed over a shorter breadth, so to get a sequence of five snapshots on a longer range of the parameter p is covered. This is an instance where the data are automatically sampled well before the tipping point compared with that for the training set. Nonetheless, we obtain correct predictions *a priori* for both out-of-distribution samples (different from training data) representing catastrophic, non-catastrophic and no transitions (see figure 5*c*,*d* and electronic supplementary material, figure S2.1 and appendix, S2), indicating the generalizability of S-EWSNet.

### 3.2. Detecting sudden transitions in empirical data using S-EWSNet

We test the robustness of S-EWSNet on available empirical data that we have access to. These data are a structural vegetation map of the Serengeti ecosystem [31] exhibiting sudden/critical transition, and the data portray the relative influences of landscape factors on the spatial heterogeneity of vegetation in the ecosystem. We show the results for Transect-2 and Transect-4 from the Serengeti ecosystem data to test the generality of our model. We particularly pick these two instances of empirical data to ensure our model results remain unaffected with reduced data length. Here, Transect-2 has a smaller number of snapshots (less than six), while Transect-4 has a higher data length prior to the transition. More details of the datasets are available in Eby *et al.* [17]. We have scaled the data to fit our model’s input size. In doing so, we first convert the binary-valued data by considering only positive values in the input stream. Next, with the obtained snapshots with different densities, we take into account the Fourier transform for each of the snapshots. The results obtained on testing S-EWSNet with empirical data are consistent with the expected results, and the ML model successfully detects and classifies the critical transition in the data prior to its occurrence. Figure 5*e*,*f* depicts the outputs of the S-EWSNet for different instances from this dataset. The results for two other transects that showed critical transition and were publicly available to us are presented in electronic supplementary material, figure S2.2 and appendix, S2). The S-EWSNet correctly predicts critical transition in both instances.

### 3.3. Robustness of trends obtained using S-EWSNet

**Figure 6.**
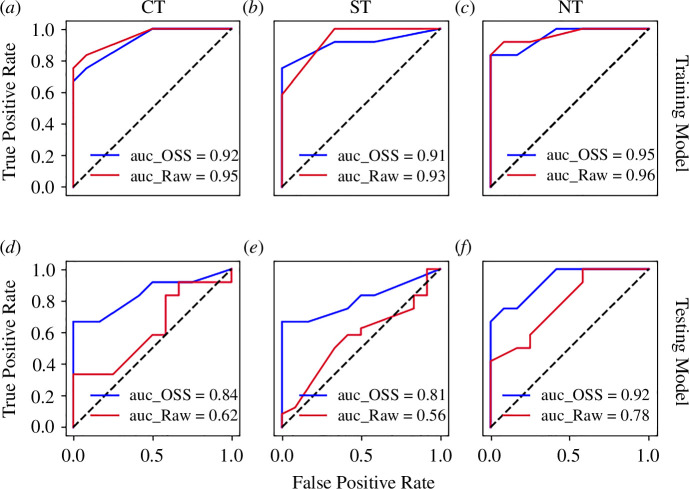
Performance of S-EWSNet on ensemble data generated from training and testing models: ROC curves exhibit the performance of S-EWSNet in predicting the three classes of transition (sudden (CT), smooth (ST) and no transition (NT)) on (*a*)–(*c*) training, and (*d*)–(*f*) testing models. The AUC estimates the goodness of the model. Higher AUC represents better performance. The black-dashed line represents the chance line where predictions are random and occur owing to chance (AUC = 0.5).

We test the reliability of S-EWSNet on a larger ensemble of both in-sample (test samples different from training but generated from the same underlying training model) data and out-of-sample (test samples generated from the testing model) data. We envision the overall predictive performance of S-EWSNet on both sets of instances using receiver operating characteristic (ROC) curves, which is a measure of the trade-off between the true positive rate (TPR) and false positive rate (FPR) of the trained model. True positive rate is the ratio of true positives to the sum of true positives and false negatives. Analogously, FPR is the fraction of false positives to the sum of false positives and true negatives. The ROC curve and the corresponding area under the curve (AUC) quantify the predictability of the classification model plotted at different classification thresholds. A higher AUC indicates a higher accuracy of the model in correctly classifying the corresponding classes. We compute the one-versus-rest ROC on 200 samples (sequence of snapshots) for each class (sudden, smooth and no transition), treating the required class as positive and the rest as negative. In figure 6*a*–*c*, we plot the ROC for each class corresponding to the training model. We observe high specificity and sensitivity for each of the classes, though the AUC is higher for no transition followed by the other classes. This indicates that a transition will always be signalled correctly, though a level of uncertainty remains in classifying the transition levels. The ROC curves for the out-of-sample data are shown in figure 6*d*–*f*. We observe similar trends as in figure 6*a*–*c*, indicating that the trained S-EWSNet is generalizable to out-of-distribution data with similar characteristic behaviour. The overall high AUC indicates reduced chances of false signals when forecasting and classifying transitions using S-EWSNet. The confusion matrices are plotted, and the metrics to evaluate the overall performance of the S-EWSNet on both the training and testing model are reported in electronic supplementary material, figure S3.1, table S3.1 and appendix, S3). Furthermore, to investigate the precedence of sampling using OSS over naive sampling, we plot the ROC curves on the raw snapshots for both the training and testing models for the same set of samples (see red curves in figure 6*a*–*f*). We observe that the model’s performance on test samples from the training data using naive sampling is comparable to that of the model when trained on data generated using OSS. However, applying the OSS considerably improves the performance of test data from a different model.

## 4. Conclusion

The impacts of critical transitions to a contrasting state are immense. Its distinction from other classes (smooth transition and no transition) is significant yet challenging. Recent research in anticipating critical transitions in temporal systems using deep learning approaches has obtained favourable outcomes [14,15], and this undoubtedly motivates the search for a deep learning-based indicator of spatio-temporal transitions. While predictions at a temporal scale are agreeable and have proven effective, there are instances where units within systems interact with their neighbours at a spatial scale. Analysing only temporal sequences may be futile in such instances, such as a change in climatic variables [32,33], cancer progression [2,3], vegetation density under grazing pressure [34,35], etc. The advent of deep learning is a bliss to the scientific community intrigued in detecting sudden transitions, waiting to be exploited further. Only a recent study by Dylewsky *et al.* [26] has developed a deep learning-based spatial indicator of climate tippings.

Here, we have developed a deep learning-based spatial indicator of critical transition, S-EWSNet by employing a novel sampling strategy (OSS) that sets it apart. While OSS is one novel approach to prevent the model from learning trends in global density change to classify transitions, building a training set that includes nonlinear driving can also suffice. However, the choice of nonlinearity could be ample; one has to identify the requisite nonlinear driving to obtain an optimal training set suited for data of varied origins. Having said that, OSS in the present framework serves our purpose of preventing overfitting and increasing the generalizability of S-EWSNet. An immediate question that arises is if OSS could provide trends for an abrupt transition, which may be preceded by a long period of stable dynamics indistinguishable from the no-transition class, which may wash out the relevant EWS information, or if one should include data that have long transients during training instead. During training, if we allow long transients in our data sampling equidistantly, and further apply max pooling, the model will miss out on distinguishable features particular to the transition time that are often present closer to the respective bifurcation, and further downsampling performed owing to max pooling may interpret the transition type incorrectly in a lot of instances as observed. On the contrary, the algorithm of OSS can overcome this issue by forcing the model to learn from the intricate features but the global density patterns. Given the model learns characteristic features particular to a transition type during training, it is able to detect transitions in test samples that may have long and short transients. As evident from our results, S-EWSNet provides an early warning indicator of critical transition and simultaneously can also discern it from other transitions as well as no transition type in out-of-sample simulated and empirical data. Instead of passing the unfiltered data directly to the model, we provide as input to S-EWSNet—clipped Fourier transforms around the lower frequency component corresponding to each snapshot along the environmental gradient. This leverages S-EWSNet from the bias-variance trade-off and overfitting of the model to the training data, which occurs if trained with sequences of raw snapshots as obtained in our preliminary experiments and adds to the generality. This novel approach of data preprocessing ensures the generality of S-EWSNet while allowing S-EWSNet to learn the attributes requisite for correctly classifying transitions and their applicability to a broad range of spatio-temporal systems. The predictions by S-EWSNet for both the simulated and empirical data agree with the ground truth labels.

Although we are limited by the lack of ample real data, we report the effectiveness of S-EWSNet based upon its reliable predictions on data obtained after stochastic simulations of a model of different origin to that of training. While we train S-EWSNet on a local positive feedback system, we test it on a system exhibiting local facilitation with explicit generating equations. Albeit we require preprocessing the data by calculating Fourier transforms, they do not interfere with the computational efficiency but rather add to the novelty and practicality of our proposed method. Computations are inexpensive and may be performed using available packages in different programming languages. Thus, S-EWSNet in its present form is an advanced deep neural network deployed as an indicator of transitions that invariably has scope for further fine-tuning suited to one’s purpose. The utility of S-EWSNet may be envisioned better with validation on more real spatial data exhibiting such transitions. It also opens up the possibility of real-time tracking of spatial climate data and ecosystem variables, spatial patterns of which carry necessary information that will aid in inferring projected risk and formulation of mitigation policies concerning such risks. Albeit the current work is geared towards the cellular-automata systems, in the future, the approach can easily be extended to other types of spatial systems manifesting different types of regular or irregular patterns [36–39].

## Data Availability

Codes and data are available in a Github repository [40] and in a Zenodo repository [41]. Supplementary material is available online [42].
